# Survival Analysis of Lymph Node Resection in Ovarian Cancer: A Population-Based Study

**DOI:** 10.3389/fonc.2020.00355

**Published:** 2020-03-19

**Authors:** Aoshuang Cheng, Jinghe Lang

**Affiliations:** Department of Obstetrics and Gynecology, Peking Union Medical College, Peking Union Medical College Hospital, Chinese Academy of Medical Sciences, Beijing, China

**Keywords:** propensity score, lymphadenectomy, ovarian cancer, prognosis, SEER

## Abstract

**Objective:** This study aimed at comprehensively investigating the survival impact of lymphadenectomy during primary surgery in ovarian cancer.

**Methods:** Based on the surveillance, epidemiology, and end results registry (SEER) database, we included ovarian cancer patients with detailed information between 2010 and 2016. Cox regression was performed to select prognostic factors. We conducted propensity score-weighted survival analysis to balance baseline variables, and series of stratified analyses to control main confounding factors.

**Results:** A total of 8,652 patients were ultimately identified. Among 4,360 patients with advanced disease, lymphadenectomy did not show significant survival benefit in general (median overall survival 44 months in non-lymphadenectomy vs. 49 months in lymphadenectomy group, *P* = 0.055). In subgroup analysis on patients received optimal debulking, lymphadenectomy did not significantly benefit the survival outcome (median overall survival 51, 47, 60, and 58 months in the non-lymphadenectomy, 1–9 lymph nodes, 10–19 lymph nodes, ≥20 lymph nodes groups, respectively, *P* = 0.287). Consistent results were observed in further stratification analyses. In optimal debulking subgroup, lymph node metastasis indicated worse survival. However, when limited the number of removed lymph nodes to more than 15, there was a marginal statistical difference in overall survival (*P* = 0.0498) while no significant difference presented in cause-specific survival (*P* = 0.129) between non-lymphadenectomy, pathological negative lymph node group and positive lymph node group. And the regions of lymph metastasis were also not significantly associate with survival (*P* = 0.123). Among 3,266 (75%) patients with apparent early-stage disease received lymphadenectomy, 7.75% of whom were reported isolated lymph nodes metastasis and have a poorer survival (*P* < 0.05).

**Conclusions:** In primary debulking for patients with advanced ovarian cancer, lymphadenectomy was not associated with more favorable outcomes when compared to no lymphadenectomy. The value of lymphadenectomy lies more in staging for apparent early disease rather than therapeutic benefit.

## Introduction

Ovarian cancer (OC) remains the most lethal cancer of the female reproductive system. Worldwide, ~230,000 women are diagnosed annually (1). About 75% of OCs are not diagnosed until the disease has progressed to advanced stage, when the 5-year relative survival rate is only 29% (2). This is in contrast to the 92% survival rate observed for early-stage disease (3). OCs represent a group of disease with different biological characteristics that shared varied histopathologic phenotypes. Epithelial ovarian carcinomas (EOC) account for ~90% of the diagnosis (4).

For primary treatment of a pelvic malignancy, the standard care consists of complete surgical staging, accurate diagnosis and primary debulking surgery (PDS) followed by adjuvant chemotherapy. No gross residual tumor (R0) after PDS is regarded as complete, which is the most important prognostic factor for survival (5–8). Based on the predominantly metastatic pattern of intraperitoneal and lymphatic spread, full pelvic, and para-aortic lymphadenectomy (LND) has been as an integral part of initial OC surgery for decades. While LND is advocated and has been widely performed, it does increase the incidence of postoperative complications. Thus, it is necessary to investigate the benefits and costs of such clinical decision.

Although retrospective studies support systematic LND in advanced OC (9–18), the results of published randomized clinical trials (RCTs) showed opposite results (19–21). With rigorous statistical method, we analyzed the updated SEER database which covers ~28% of the U.S. population to provide further and complementary real-world evidence to investigate the role of lymph node resection in OC, especially in advanced OC.

## Materials and Methods

### Data Source and Variables

With granted access, the ASCII-encoded text file published in April 2019 with treatment recodes was downloaded from SEER website of the National Cancer Institute (http://seer.cancer.gov). In this version of raw data, the new diagnosis was included until the end of 2015, and patients were followed to December 31, 2015. According to SEER documentation (https://seer.cancer.gov/data-software/documentation/ascii-files.html), we interpreted all 147 coded variables to their actual descriptions, and then abstracted relevant variables: primary site (ICD-O-2), the number and sequence of all reportable malignant, age at diagnosis, year at diagnosis, race, histological cell type, grade, tumor size, lateral information, stage (6th AJCC, available after 2004), chemotherapy recode, operation methods, residual disease status (available after 2010), lymph node status, regions of lymph node involvement status, and insurance recode. In the SEER database, the information on chemotherapy recoded whether or not chemotherapy was definite performed, however the detailed regimen and cycle was unavailable.

Additionally, the 6th AJCC staging of OC in the SEER recode was converted to the International Federation of Gynecology and Obstetrics 2014 staging system (FIGO 2014). We divided the extent of lymphadenectomy according to the number of lymph nodes (LN) removed, into four groups of 0 LN (non-LND), 1–9 LNs, 10–19 LNs, and ≥20 LNs. Lymph node metastatic status was evaluated using the lymph node ratio (LNR) calculated by dividing the number of positive pathological lymph nodes to total number of lymph nodes removed.

### Cohort Selection

**Figure 1:**
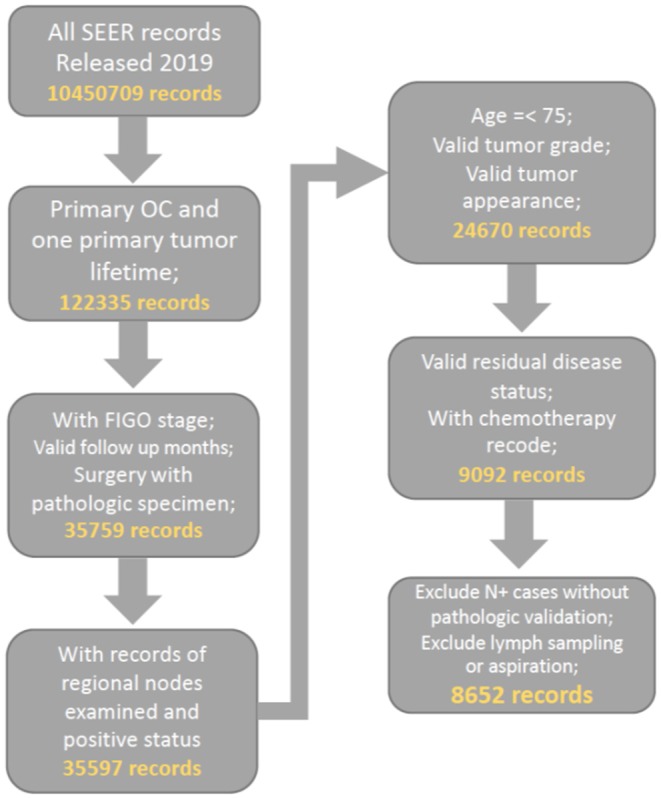
Flow chart of data selection.

Patients who were diagnosed with primary malignant ovarian cancer (ICD-O-2, C569) and had complete surgery recodes, FIGO stage and valid follow up recode were preliminarily included. We further filter the cohort by our quality control criteria of valid and complete recodes on tumor size, number of lymph node removed, lymph node status, and residual disease status. Consequently, all included patients were diagnosed from 2010 to 2016 as the important variable of residual disease status was started collecting in SEER database after 2010. The patient age limit was 75 years to reduce selection bias. Patients with recodes for lymph node sampling and aspiration were excluded. The detailed selection procedure is summarized in Figure 1. To ensure the reliability of the included data, we randomly selected 1,000 cases and compared them with information from official SEER^*^STAT software (https://seer.cancer.gov/seerstat/), which proved the consistency of our data.

Patients with presumed early disease were defined by 6th AJCC-T1 or T2, and AJCC-M0 (FIGO I-IIIA1), including those with confined tumor and positive lymph node (FIGO IIIA1). In survival analysis of advanced OC patients (FIGO III-IV), we excluded the patients with the FIGO-IIIA1 disease, and limited surgical method to cytoreductive surgery (recoded debulking or pelvic exenteration).

### Statistical Analyses

The outcome was evaluated using both overall survival (OS) and cause-specific survival (CSS). A Cox regression model was used to assess the prognostic effect of multiple variables. Due to the imbalance in baseline variables between multinomial groups, we adopted a weighted strategy to obtain equivalent arms for comparative analysis, PSW (propensity score weighted) was selected for subsequent analyses (22) (Supplementary Figure 1). The survival comparison between weighted groups was calculated by the weighted log-rank test according to Kevin Andrew (23). A *P* < 0.05 was considered to be significant. Data analysis and plot generation were performed in R (version 3.6.1).

## Results

### Patients and Characteristics

After quality control and filter, 8,652 patients were finally included. The median patient age was 57 years and median follow-up was 43 months. Among 8,652 included patients, 5,650 (65.3%) patients received LND with an average of 15 lymph nodes and 1,492 (26.4%) of them had at least one pathologically positive lymph node. While almost three-quarters of the patients with early stage OC received LND, half of the patients with advanced OC underwent LND. The younger and early stage patients were more likely to have a lymphadenectomy (Patients under 60 was 69.1 vs. 59.8% for the elder; Patients with early OC was 74.5 vs. 42.8% for patients with advanced OC). As LND was more commonly performed in R0 surgery than in nonR0 surgery (72.1 vs. 55.7%), the extent of LND was associated with the rationality of surgery. Characteristics of patients at baseline according to the number of nodes resected was summarized in Table 1.

**Table 1:** Characteristics of patients at baseline according to the number of nodes resected.

		**Total (%)**	**Non LNs (*n* = 2,490)**	**LNDs (*****n*** **=** **5,650)**			***p***
				**1–9 LNs (*n* = 2,371)**	**10–19 LNs (*n* = 1,770)**	**≥20 LNs (*n* = 1,563)**	
Age	40–60	4,491 (52.28%)	1,316	1,233	1,004	938	ref
	>60	3,090 (35.97%)	1,241	806	581	462	<0.001
	≤40	1,009 (11.75%)	383	278	185	163	<0.001
Race	White	6,973 (81.18%)	2,349	1,894	1,435	1,295	ref
	Asian or Pacific Islander	964 (11.22%)	313	240	222	189	0.1785
	Black	555 (6.46%)	246	154	92	63	<0.001
	American Native	52 (0.61%)	16	18	12	6	<0.001
	N.A.	46 (0.54%)	16	11	9	10	0.927
Tumor size	≤5 cm	1,991 (23.18%)	856	474	328	333	ref
	5–10 cm	2,582 (30.06%)	899	711	512	460	<0.001
	10–20 cm	3,246 (37.79%)	951	910	730	655	<0.001
	>20 cm	715 (8.32%)	210	210	185	110	<0.001
	N.A.	56 (0.65%)	24	12	15	5	0.133
Lateral	Unilateral	5,404 (62.91%)	1,599	1,496	1,242	1,067	ref
	Bilateral	3,127 (36.4%)	1,310	808	517	492	<0.001
	NOS	59 (0.69%)	31	13	11	4	0.001
Grade	I	1,166 (13.57%)	386	313	266	201	ref
	II	1,588 (18.49%)	476	433	369	310	0.252
	III	3,257 (37.92%)	1,133	851	661	612	0.204
	IV	2,579 (30.02%)	945	720	474	440	<0.001
Histopathology	HGSOC	3,634 (42.31%)	1,418	969	655	592	ref
	LGSOC	588 (6.85%)	228	154	104	102	0.935
	Mucinous	730 (8.5%)	237	198	169	126	0.001
	Endometrioid	1,177 (13.7%)	268	326	309	274	<0.001
	Clear-cell	761 (8.86%)	160	202	200	199	<0.001
	EOC	1,158 (13.48%)	350	322	254	232	<0.001
	Germ malignancy	272 (3.17%)	163	64	30	15	<0.001
	SS malignancy	98 (1.14%)	44	34	13	7	0.025
	Malignancy (NOS)	67 (0.78%)	32	13	15	7	0.198
	Others	105 (1.2%)	40	35	21	9	0.126
Stage	I	3,333 (38.8%)	872	877	854	730	ref
	II	923 (10.75%)	214	247	250	212	0.316
	III	3,128 (36.41%)	1,261	855	515	497	<0.001
	IV	1,206 (14.04%)	593	338	151	124	<0.001
Surgery method	Debulking	3,582 (41.7%)	1,431	966	628	557	ref
	BSO&Ome&hys	2,530 (29.45%)	507	755	668	600	<0.001
	USO_nonhys	358 (4.17%)	224	69	49	16	<0.001
	BSO_nonhys	195 (2.27%)	92	35	43	25	0.011
	BSO&hys	988 (11.5%)	361	240	204	183	0.005
	others	782 (9.1%)	299	219	151	113	0.499
	Pelvic exenteration	155 (1.8%)	26	33	27	69	<0.001
Residual disease	R0	5,289 (61.57%)	1,478	1,402	1,247	1,162	ref
	Non R0	1,742 (20.28%)	936	474	178	154	<0.001
	No debulking	1,559 (18.15%)	526	441	345	247	<0.001

*LGSOC, low-grade serous ovarian cancer; HGSOC, high-grade serous ovarian cancer; EOC, epithelial ovarian cancer; SS Malignancy, sex cord stromal malignant tumors; Germ Malignancy, malignant germ cell tumor; NOS: not available; USO, unilateral oophoro-salpingectomy; BSO, bilateral oophorosalpingectomy; Hys, hysterectomy; Ome, omentectomy; ref, reference*.

### Multivariate Survival Analysis

**Figure 2:**
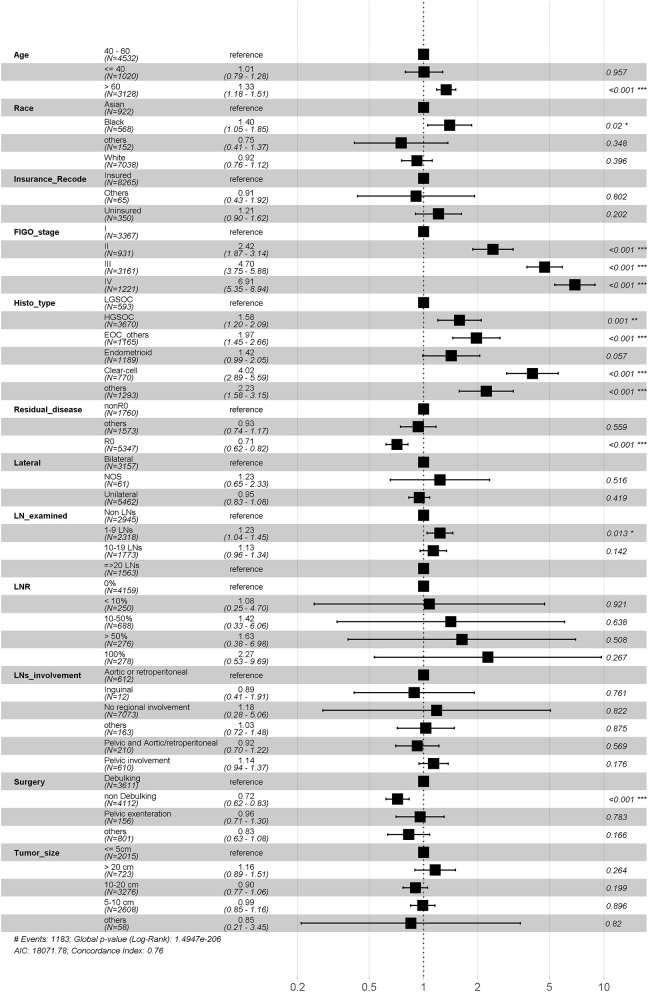
Multivariate survival analysis. LN, lymph node; LNR, positive lymph node ratio.

We performed multivariate cox regression in all of included patients to assess prognostic factors. The age, race, insurance, stage, grade, histopathology, residual disease status, extent of surgery and LNR were all found related with overall survival. Notably, while the number of LN examined was not significantly associated with prognosis (1–9 LNs to Non LNs: HR 0.984, *P* = 0.978; 10–19 LNs to Non LNs: HR 0.91, *P* = 0.72; ≥20 LNs to Non LNs: HR 0.80, *P* = 0.414), the increased LNR shown unfavorable effects on overall survival (0% as reference, the HR for <10, 10–50, >50, and 100% group are 0.97, 1.29, 1.51, and 2.08, respectively) (Figure 2). The similar trend was observed when we performed multivariate cox regression in advanced disease (Supplementary Figure 3). Additionally, the number of LN examined was found significant prognosis factor (1–9 LNs to Non LNs: HR 0.78, *P* = 0.074; 10–19 LNs to Non LNs: HR 0.67, *P* = 0.005; ≥20 LNs to Non LNs: HR 0.51, *p* < 0.001) (Supplementary Figure 2).

To eliminate possible confounders, the other identified prognostic variables, such as FIGO stage, surgery method, histopathology, tumor size, and residual disease were included in PSW method as adjusted variables or used in subgroup analysis as stratified variables.

### Lymphadenectomy in Advanced OC

**Figure 3:**
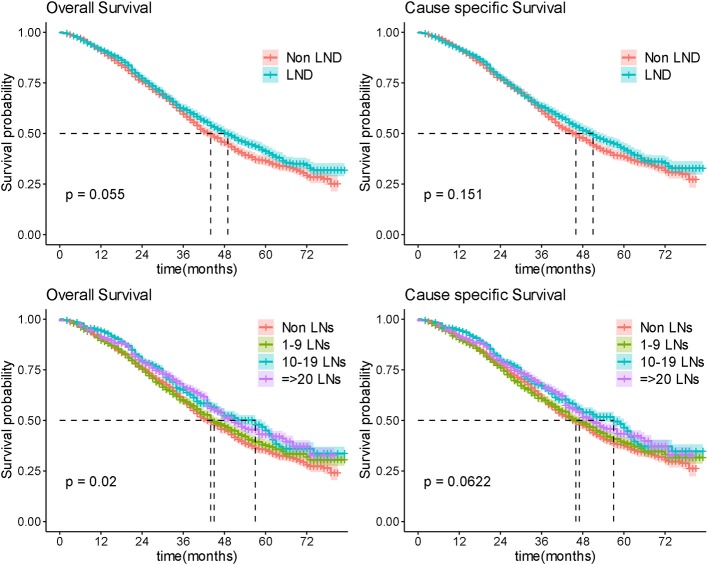
Overall survival by lymphadenectomy. LN, lymph node.

Among the 4,107 (47.5%) patients with advanced OC, LND was performed in 2,253 (54.8%) patients and of whom 1,241 (55.1%) were found to have pathologically positive lymph nodes. There was a marginal statistical difference in OS (Median OS 44 vs. 49 months; *P* = 0.055) while no significant difference in CSS (Median CCS 46 vs. 51 months; *P* = 0.151) between non-LND and LND groups (Figure 3) in general. Stratification analysis on histopathologic type, FIGO stage, and residual disease status were conducted to control confounding factors, respectively.

**Figure 4:**
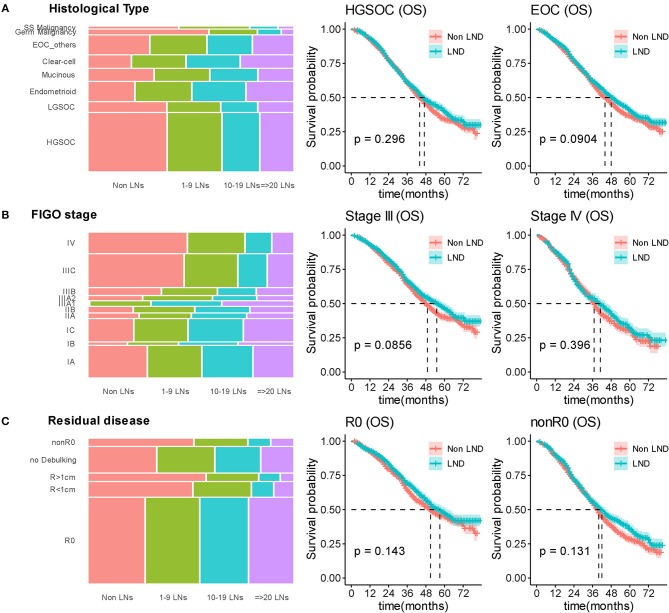
Stratified survival analyses by lymphadenectomy. **(A)** Distributed relationship between number of removed lymph nodes and histological type; Kaplan-Meier curves for overall survival in stratification analysis according to histological type. **(B)** Distributed relationship between number of removed lymph nodes and stage; Kaplan-Meier curves for overall survival in stratification analysis according to stage. **(C)** Distributed relationship between number of removed lymph nodes and residual disease status; Kaplan-Meier curves for overall survival in stratification analysis according to residual disease. SS Malignancy, sex cord stromal malignant tumors; Germ Malignancy, malignant germ cell tumor; LN, lymph node; LND, lymphadenectomy.

As the primary histopathological type, we limited the filter criteria to EOC, and observed 3,902 (95.0%) patients were diagnosed with EOC. The median OS was 44 and 48 months (*P* = 0.0904) in the non-LND and LND groups. Among those with EOC, the patients with high-grade serous ovarian carcinoma (HGSOC) account for 71.3% (2,783/3,902), and there was also no significant difference in survival between the two groups (*P* = 0.296) (Figure 4A).

In stratification analysis according to FIGO stage, 2,895 (70.5%) patients were diagnosed with FIGO-III OC, the median OS was 49 and 55 months in the non-LND and LND groups (*P* = 0.0856). For other 1,212 (29.5%) patients with FIGO-IV stage, statistical difference between the two groups furtherly reduced (*P* = 0.396) (Figure 4B).

As residual disease status was important prognostic factor, we stratified analysis according to residual disease. For patients receiving R0 surgery, the median OS was 51 and 57 months in the non-LND and LND groups, respectively (*P* = 0.143; Figure 4C). Other patients underwent suboptimal cytoreductive surgery (nonR0), the median OS was 37 and 39 months (*P* = 0.131; Figure 4C) in the non-LND and LND groups.

**Figure 5:**
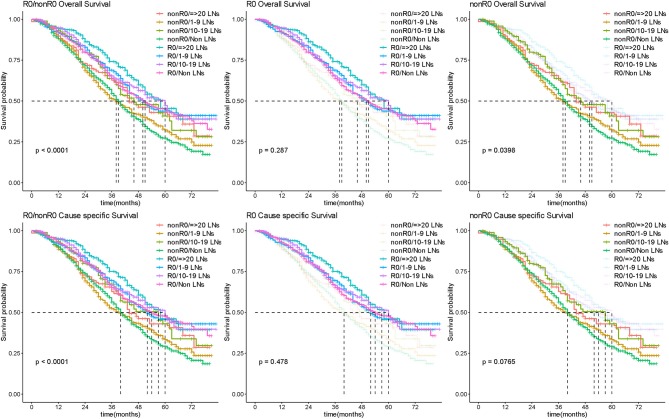
Survival outcome by extent of lymphadenectomy. Kaplan-Meier curves for overall and cause-specific survival analysis according to the number of removed lymph nodes for R0 and nonR0 surgery in patients with advanced OC. LN, lymph node.

Extent of LND was observed in line with radicality of surgery which was of important confounding factor. We further analyzed the effect of different extent of LND on survival to explore this issue. As there was still no significant difference in extent of LND subgroup comparisons (the median OS was 51, 50, 60, and 58 months in non-LND 1–9 LNs, 10–19 LNs, and ≥20 LNs groups, respectively, *P* = 0.287), no benefit was observed in more radical LND group for patients with R0 surgery (Figure 5; Supplementary Figure 4). In patients of nonR0 group, more radical LND increased the OS outcome (*P* = 0.0398; Figure 5), but difference presented no significance in CSS comparison (*P* = 0.0765; Figure 5).

### Role of Lymph Node Metastasis on R0 Surgery

**Figure 6:**
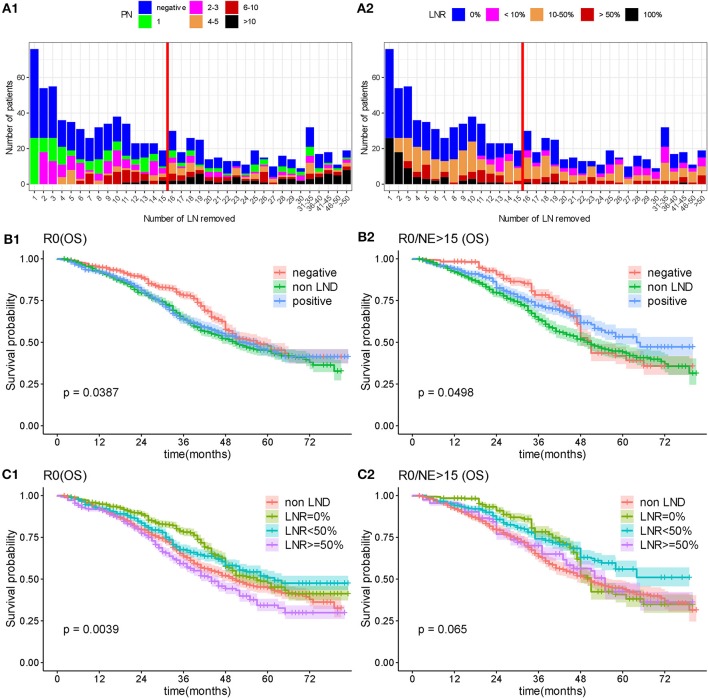
Overall survival by lymph node status. **(A)** Distributed relationship between number of removed lymph nodes and involved lymph nodes. **(B)** Overall survival analysis according to lymphadenectomy and status of removed lymph nodes. **(C)** Overall survival by lymphadenectomy and lymph node ratio. PN, number of positive lymph node; NE, number of removed lymph node; OS, overall survival; LNR, lymph node ratio.

As the operation of LND theoretically removed positive lymph nodes to benefit prognosis, we furtherly evaluated whether radical LND to remove disease in the lymphatics brought ultimate survival benefit in patients underwent R0 surgery. We firstly compared the survival outcome surgery between non-LND, negative LN and positive LN groups, the results showed difference on OS (*P* = 0.0387; Figure 6B1), while there was no significant difference in CCS (*P* = 0.119; Supplementary Figure 5). Analysis on LNR showed that patients with more pathologically positive for disease had a significantly worse prognosis relative to patients with lymph nodes that were negative for disease (the median OS was 51, 57, 62, and 44 months in non-LND, LNR = 0%, LNR < 50%, and LNR ≥ 50% group, respectively, *P* = 0.0039; Figure 6C1).

As the lymph nodes involvement status was heterogeneous, which depends on multiple factors, including the number of removed nodes, the bias of the surgeon, and the pathologist's technical approach for analyzing lymph nodes, we limited the number of removed lymph nodes to more than 15, which were considered to be an exhaustive LND. Then the statistical difference in OS between non-LND, negative LN and positive LN groups reduced, and no significant difference presented in CSS (Median OS: 50, 51, and 64 months, respectively, *P* = 0.0498, Figure 6B2; Median CSS: 54, 51, and 65 months, *P* = 0.129, Supplementary Figure 5). In detailed comparison on LNR, there was no significant difference between non-LND, LNR = 0%, LNR < 50%, and LNR ≥ 50% groups (*P* = 0.065 in OS, Figure 6C2; *P* = 0.162 in CSS, Supplementary Figure 5). Although no statistical difference, it was a remarkable trend that with the increasing number of removed lymph nodes, the survival of positive lymph node group tended to be better while the negative lymph node group become worse (Figure 6; Supplementary Figure 5).

**Figure 7:**
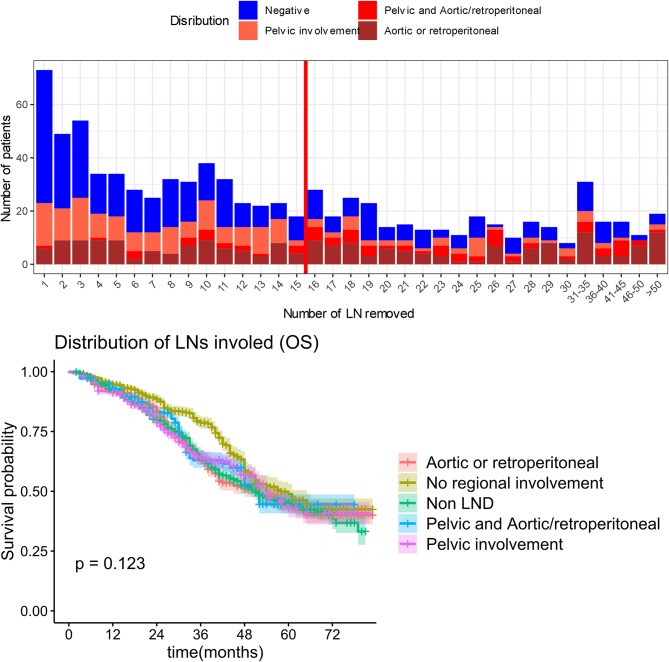
Overall survival by lymphadenectomy and lymph node metastasis region.

Additionally, we analyzed the regions of the lymph metastases including non-LND, no regional involvement, para-aortic lymph node involvement, pelvic lymph node involvement, or both of pelvic and para-aortic lymph node involvement, and the median OS was 51, 58, 52, 54, and 51 months, respectively (*P* = 0.123). The involved regions were not significantly associated with survival prognosis (Figure 7).

### Survival of Early Stage OC

**Figure 8:**
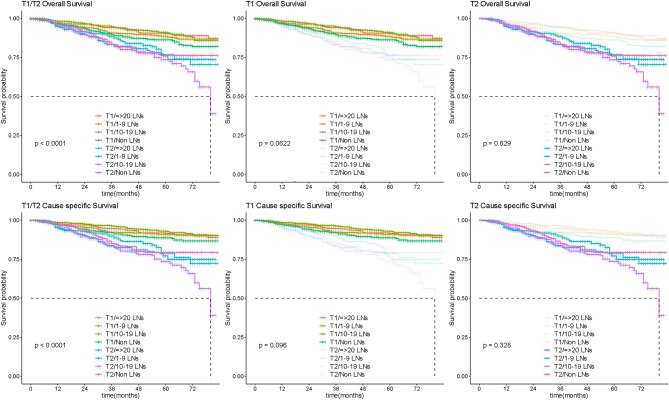
Survival by lymphadenectomy in early OC. Overall and cause-specific survival analysis according to the number of lymph nodes resected for patients with apparent early stage disease. LN, lymph node.

**Figure 9:**
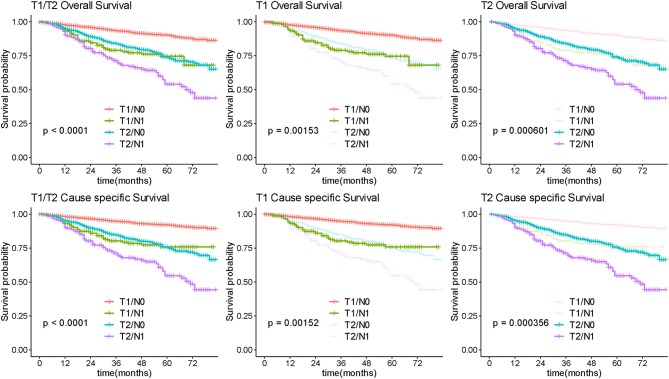
Survival analysis by isolated lymph node status. Overall and cause-specific survival analysis according to lymph node involvement for patients with early stage ovarian.

Among 4,545 patients with apparent early-stage disease, LND was performed in 3,459 (76.1%) of them. After stratification analysis according extent of primary tumor invasion, the number of removed lymph nodes was not associated with survival prognosis in patients with apparent confined disease (Figure 8). Additionally, 253 (7.31%) were upstaged to FIGO-IIIA1 due to isolated positive lymph node. These patients had significantly poorer survival than patients with negative lymph nodes (Figure 9).

## Discussion

Dissection of pelvic and para-aortic lymph nodes is widely performed as an integral part of comprehensive staging laparotomy or cytoreductive surgery for patients with OC. However, this operation remains debatable because of high operative complication. The core issue of the controversy is whether and when the removal could improve survival.

Many retrospective studies (9–18) have demonstrated survival benefits of LND in late OC.

Using SEER database, Chan et al. (16) and Rouzier et al. (18) showed survival benefits when more nodes were resected in all stages of OC. Their analysis was limited by the deficiency of important variables due to the unavailability of some records in SEER before 2004, including the volume of residual disease. This limitation affected their conclusions because, as we presented, the residual disease is an essential stratified variable in analysis. Additionally, in light of the advances in treatment, survival of OC patients improved over time (24), we included contemporary OC patients that diagnosed since 2010 when the carboplatin and paclitaxel were widely used in first-line treatment. Moreover, by employing reasonable statistical method of PSW, we adjusted the balance of baseline in survival comparison, which enhanced representativeness of groups and improved intergroup comparability.

In the recently published RCT of LION (20), researchers included patients with advanced OC (FIGO IIB-IV) who had normal appearance lymph nodes. They observed that systematic LND was not associated with longer overall or progression-free survival (PFS) than non-LND, while cause higher incidence of postoperative complications. This trial finally included 647 (34.1%) patients from preliminarily 1,895 patients and 203 patients were excluded due to intraoperative bulky lymph nodes. However, the including criterion that intraoperative classification of lymph nodes by the surgeon was not definite, which would result in potential evaluator bias (25). Therefore, results from real-world study is needed, and the population-based analysis provided further evidence to investigate the controversy.

In our results, LND was performed in 2,378 (56.8%) patients of advanced OC. The analyses indicate that LND did not significantly increase CSS or OS, and the extent of LND was not significantly associated with survival outcome either. After stratified analyzing according to latent confounding factors, respectively, the statistical difference between LND and non-LND groups became reduced. Moreover, while the survival outcome significantly differed between R0 and nonR0 group, the insignificant statistical difference in survival between different extent of LND within R0 subgroup is consistent with the concept that an optimal removal of gross residual disease is the most crucial factor in surgery treatment, and a radical LND did not significantly improve ultimate survival as tumor cytoreduction.

As the theoretical aim of LND in surgery is at detecting positive lymph nodes to remove microscopic metastasis, we furtherly explored the survival impact of removing pathological lymphatic metastasis in patients with no gross residual disease. Published studies evaluated the lymph metastasis status with the number of positive nodes, LNR, or log odds of positive lymph nodes (LODDS) and reported that the extent of lymph node involvement was a prognostic factor in OC (26–29). In RCT (20), the researchers did not further analysis the patients who was found pathologic positive lymph nodes. It can be inferred from our results, patients with more lymphatic metastasis had worse prognosis, however, with the increasing number of removed lymph nodes, the survival of positive lymph node group tended to be better while the negative lymph node group become worse, which probably due to negative effect result from radical LND. Consequently, positive lymph node status might be a prognostic predictive factor to evaluate the invasiveness and progression of the disease, but radically removing latent pathological metastasis in lymphatics might not show significant promotion to ultimate survival outcome in debulking surgery.

There are several explanations for the few survival benefits of lymphadenectomy. On one hand, although radical LND could remove latent microscopic lymphatic metastasis, this practice shows a minor contribution as compared to optimal debulking surgery combined chemotherapy. On the other hand, non-negligible risk of surgery-related complications and morbidity have to be taken into consideration in the clinic, especially for patients with advanced OC. Retroperitoneal surgery would have an impact on the postoperative course and long-term complications (20). Moreover, some studies suggested the metastasis of lymph nodes was suggested to have had an indolent evolution (30–35), while related studies are limited.

Analysis in early-stage patients showed LND was not significantly associated with overall prognosis. However, among groups of LND, a small proportion of the patients (7.75%) with isolated positive lymph node were upstaged to advanced stage after LND, and these patients had a significantly poorer prognosis than others. For presumed early-stage patients, LND is a crucial surgical step during staging procedures and could identify patients with high-risk.

In current study, we emphasize the importance of rigorous methodologies and reasonable statistical treatment in retrospective analysis. Our study complemented existing RCTs with further analyses and large data from real-world. However, there are several weaknesses that must be acknowledged. We were not able to compare the operative procedures and their impact in a stricter manner due to the lack of detailed information regarding preoperative assessment and postoperative complications. The absence of a detailed description of chemotherapy program, regimen and cycle, targeted therapy, and patterns of recurrence would also limit the analysis. In addition, the median follow-up was 43 months, which is probably a short period to better determine the survival.

## Conclusion

Our results indicate that in patients with advanced ovarian cancer, systematic pelvic and paraaortic lymphadenectomy was not associated with better outcomes when compared to no lymphadenectomy. The value of lymphadenectomy lies more in staging for apparent early disease rather than therapeutic treatment in ovarian cancer.

## Data Availability Statement

The datasets analyzed in this study can be found in the SEER website of the National Cancer Institute (http://seer.cancer.gov).

## Author Contributions

JL conceived of the original idea for the study, interpreted results, edited the paper, and was overall guarantor. AC obtained ethical approval, contributed to the preparation of the data set, carried out the statistical analysis, interpreted results, and contributed to drafts of the paper.

### Conflict of Interest

The authors declare that the research was conducted in the absence of any commercial or financial relationships that could be construed as a potential conflict of interest.

## Supplementary Material

Supplementary Figure 1 Comparison of statistical methodology. **(A)** The original baseline. **(B)** Adjusted baseline by propensity score weighted.

Supplementary Figure 2 Multivariate survival analysis on patients of early-stage OC.

Supplementary Figure 3 Multivariate survival analysis on patients of advanced OC.

Supplementary Figure 4 Stratified cause-specific survival analyses by lymphadenectomy according histological type, stage, and residual disease, respectively.

Supplementary Figure 5 Cause-specific survival analyses survival by lymphadenectomy and lymph node status.
