# Supplementary figures and images for: Survival Analysis of Lymph Node Resection in Ovarian Cancer: A Population-Based Study
Source: Front Oncol. 2020 Mar 19;10:355. doi: 10.3389/fonc.2020.00355 (PMC7096485; doi:10.3389/fonc.2020.00355)

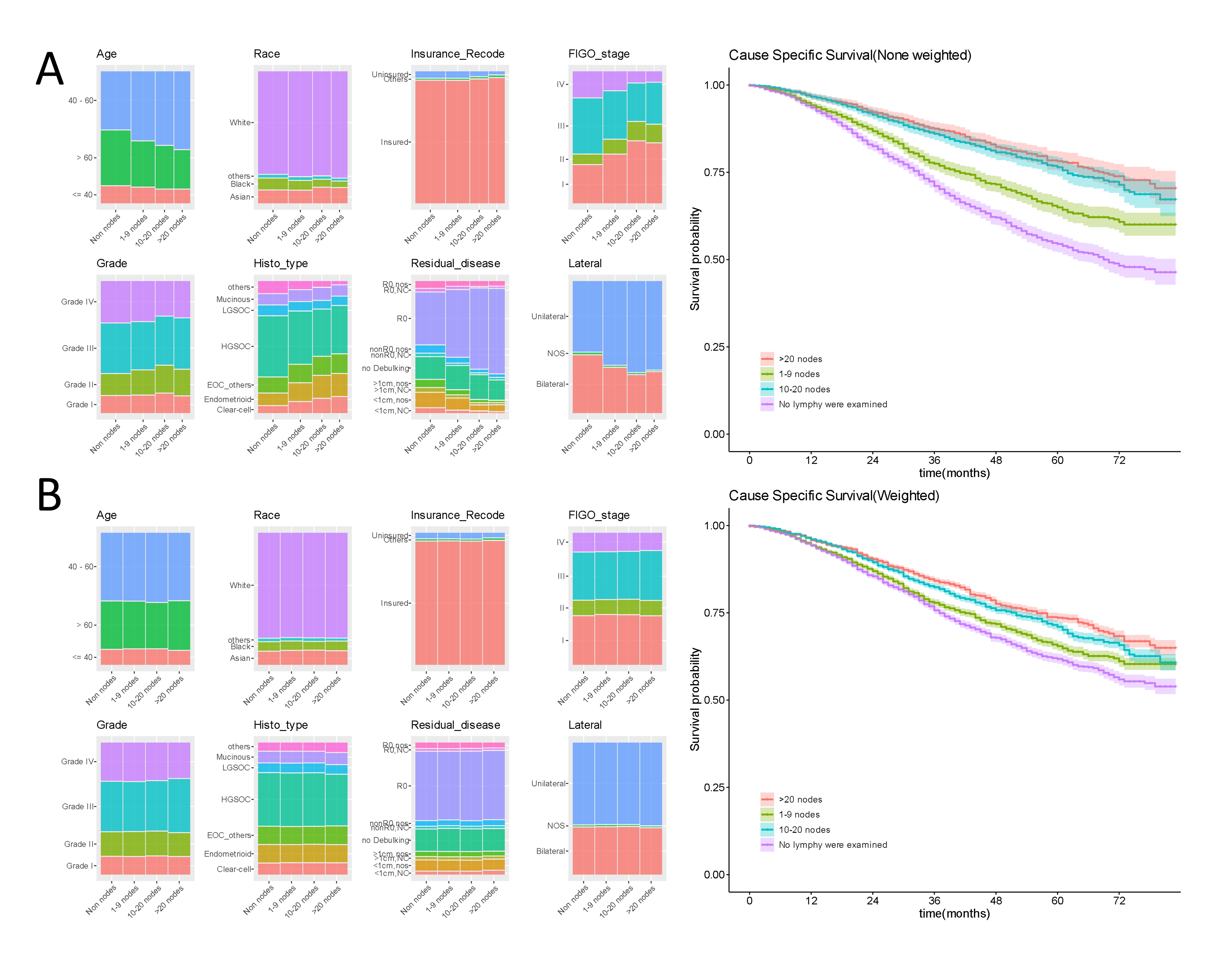

Supplement: Supplementary Figure 1 — Comparison of statistical methodology. (A) The original baseline. (B) Adjusted baseline by propensity score weighted. [file Image_1.TIF]

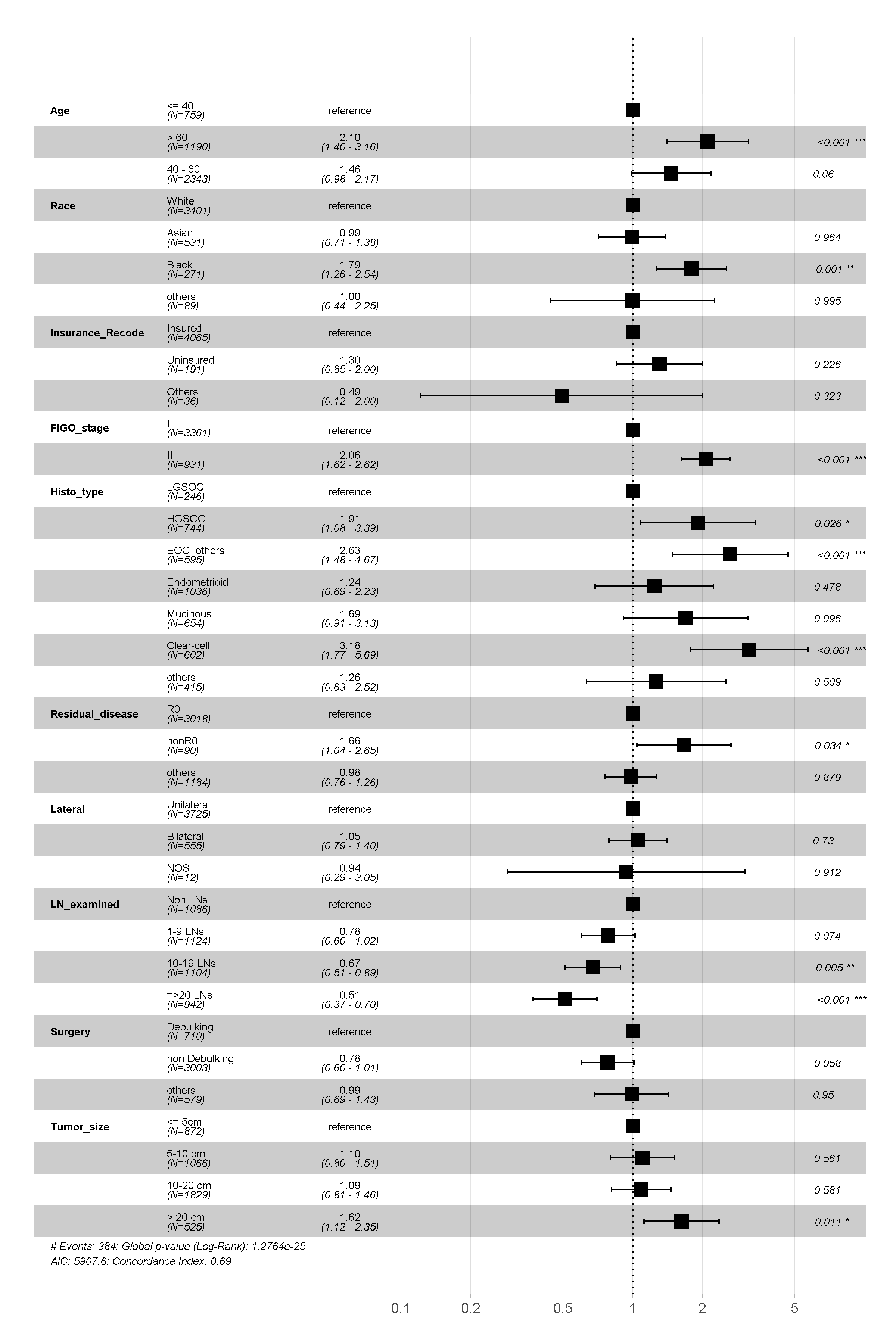

Supplement: Supplementary Figure 2 — Multivariate survival analysis on patients of early-stage OC. [file Image_2.TIFF]

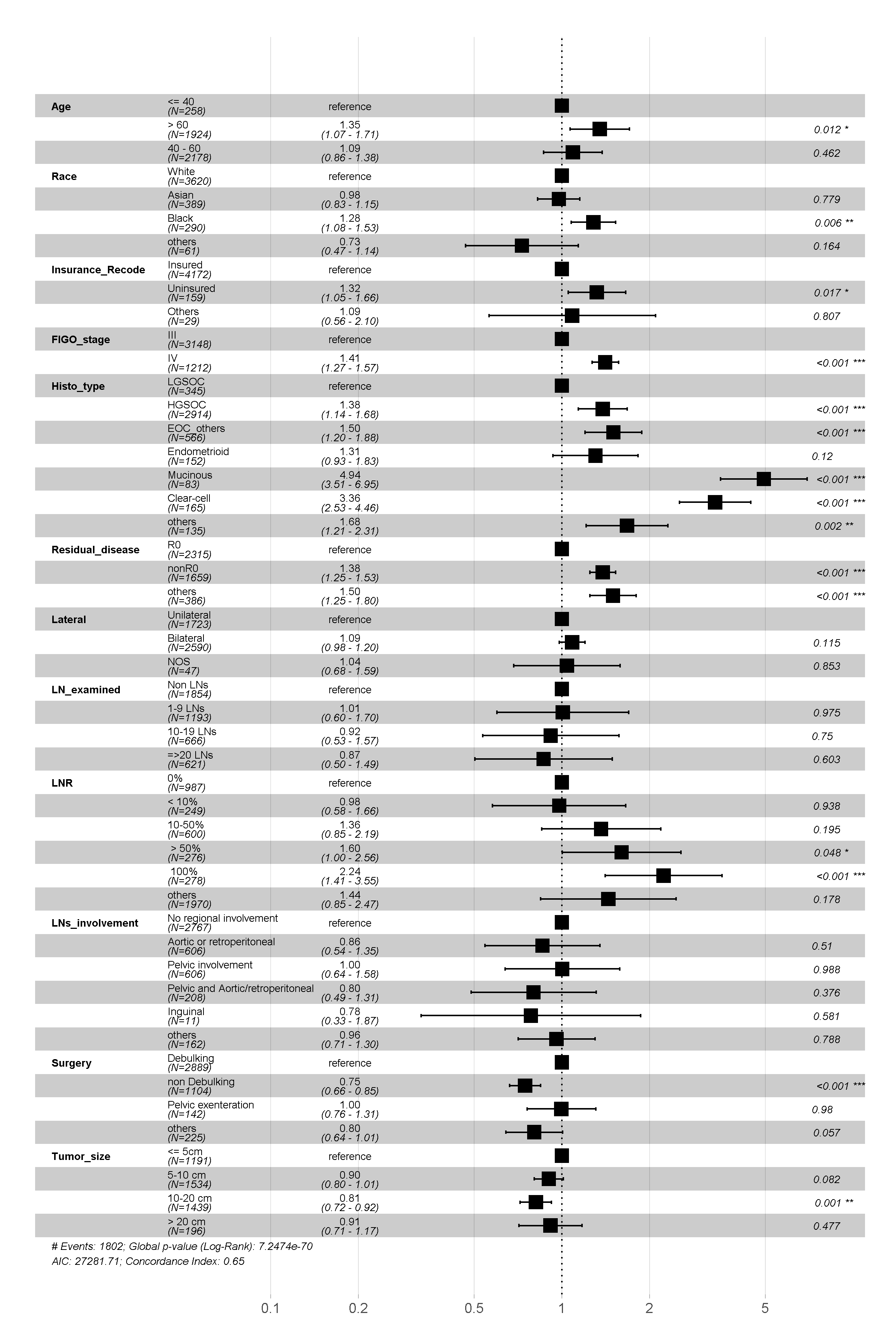

Supplement: Supplementary Figure 3 — Multivariate survival analysis on patients of advanced OC. [file Image_3.TIFF]

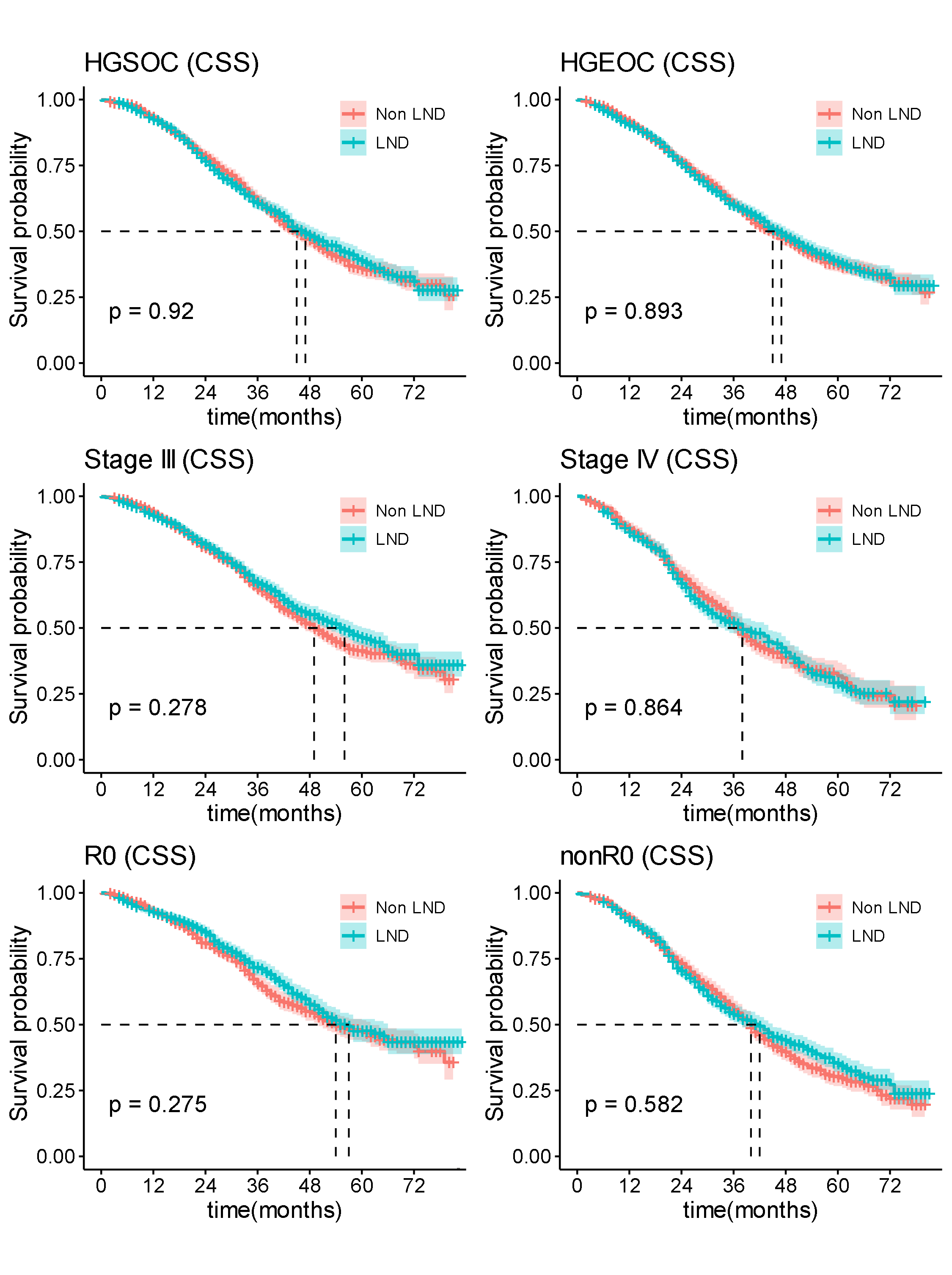

Supplement: Supplementary Figure 4 — Stratified cause-specific survival analyses by lymphadenectomy according histological type, stage, and residual disease, respectively. [file Image_4.tif]

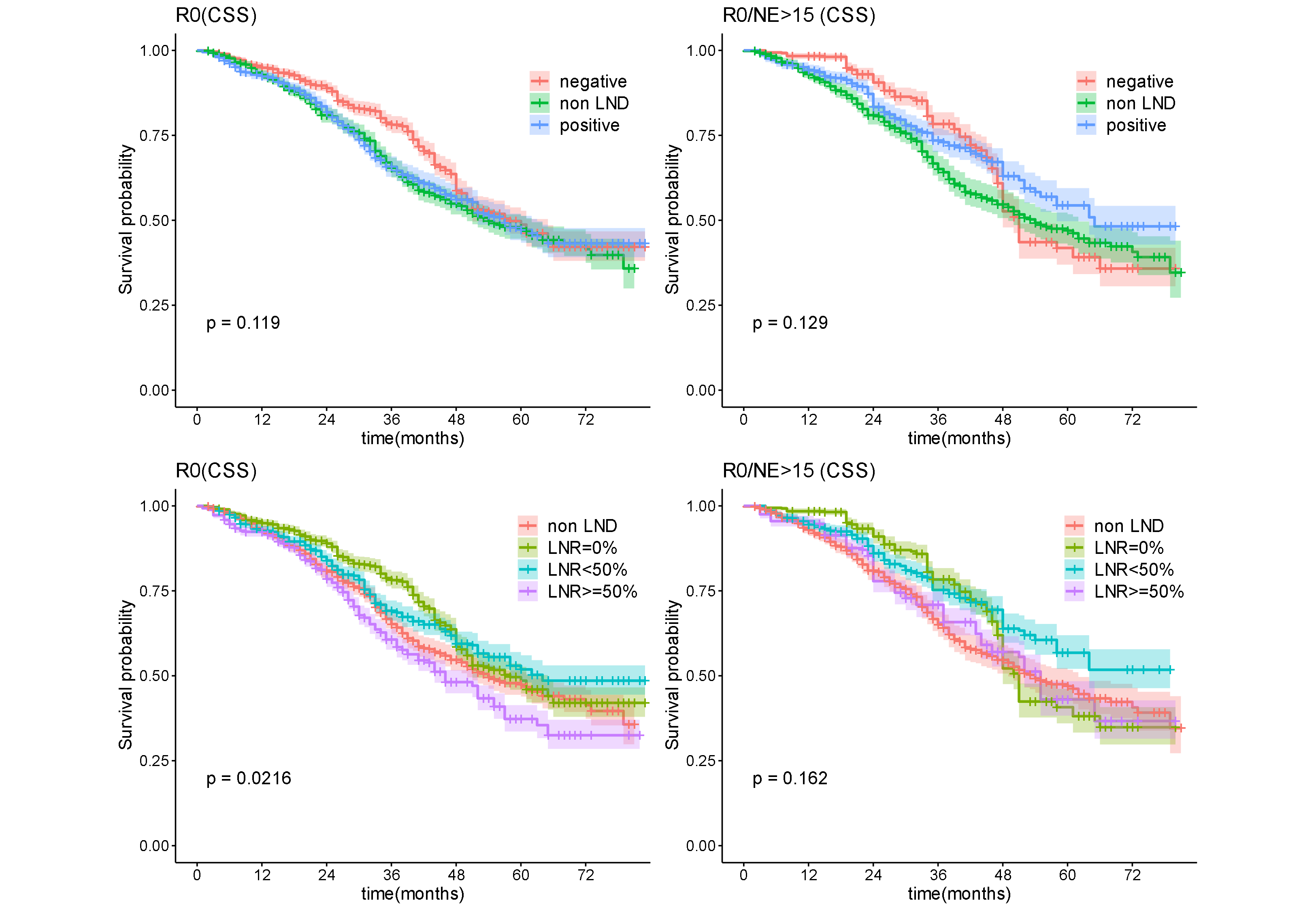

Supplement: Supplementary Figure 5 — Cause-specific survival analyses survival by lymphadenectomy and lymph node status. [file Image_5.tif]
